# Development and validation of a prediction model to estimate the risk of liver cirrhosis in primary care patients with abnormal liver blood test results: protocol for an electronic health record study in Clinical Practice Research Datalink

**DOI:** 10.1186/s41512-019-0056-7

**Published:** 2019-05-23

**Authors:** Suvi Härmälä, Alastair O’Brien, Constantinos A. Parisinos, Kenan Direk, Laura Shallcross, Andrew Hayward

**Affiliations:** 1 Institute of Health Informatics, University College London, 222 Euston Road, London, NW1 2DA UK; 2 Division of Medicine, University College London, Rayne Building, 5 University Street, London, WC1E 6JJ UK; 3 Institute of Epidemiology and Health Care, University College London, 1-19 Torrington Place, London, WC1E 7HB UK

**Keywords:** Prediction model, Prognosis, Cirrhosis, Liver disease, Primary care, Electronic health records, Public health, Prevention

## Abstract

**Background:**

Driven by alcohol consumption and obesity, the prevalence of non-viral liver disease in the UK is increasing. Due to its silent and slow nature, the progression of liver disease is currently unpredictable and challenging to monitor. The latest National Institute for Health Care Excellence cirrhosis guidelines call for a validated risk tool that would allow general practitioners to identify patients that are at high risk of developing cirrhosis.

**Methods:**

Using linked electronic health records from the Clinical Practice Research Datalink (a database of > 10 million patients in England), we aim to develop and validate a prediction model to estimate 2-, 5- and 10-year risk of cirrhosis. The model will provide individualised cirrhosis risk predictions for adult primary care patients, free from underlying liver disease or viral hepatitis infection, whose liver blood test results come back abnormal. We will externally validate the model in patients from 30 further Clinical Practice Research Datalink general practices in England.

**Discussion:**

The prediction model will provide estimates of cirrhosis risk in primary care patients with abnormal liver blood test results to guide referral to secondary care, to identify patients who are in serious need of preventative health interventions and to help reassure patients at low risk of cirrhosis in the long term.

## Background

Cirrhosis secondary to liver disease usually develops slowly over many years. Liver disease is often asymptomatic and its presence unknown to the patient until the liver function becomes so severely compromised that the patient develops life-threatening complications such as gastrointestinal bleeding, jaundice and abdominal infections. The only cure for advanced cirrhosis is liver transplant, and without a new liver, only half of the patients survive past 5 years from the diagnosis [1]. Worryingly, in the UK, driven by the upward trend in obesity and alcohol consumption, the prevalence of non-viral liver disease and those who develop cirrhosis is increasing [2], and whereas death rates in many other chronic diseases such as cardiovascular disease have improved over the last three decades, the number of premature deaths from cirrhosis continues to rise [3].

Many of the known drivers of liver damage, such as alcohol consumption and obesity, are modifiable. The earlier the damage is known, the greater the potential for behaviour change interventions and other preventative treatment to be effective and limit or even halt the progression of the disease. Due to its silent and slow nature, however, the progression of liver disease is currently unpredictable and challenging to monitor and thus identifying the target population for these interventions is currently not possible. The latest National Institute for Health Care Excellence (NICE) guideline for assessment and management of cirrhosis calls for a validated risk tool that would allow general practitioners (GPs) to identify patients who are at high risk of developing cirrhosis [4].

The first indicator of some form of liver damage is often an abnormal liver blood test result, and this could signal the need to refer to secondary care for further investigations or present an opportune timing for a preventative health intervention. However, while liver blood tests are the third most commonly ordered test in primary care since 2001 [5] and potentially over 20% of patients tested have an abnormal result [6], only very few, possibly less than 5% of patients, are diagnosed with liver disease during the following 2 years [7]. On the other hand, as many as 50% of the cirrhosis cases develop unnoticed and are first identified only during an emergency hospitalisation due to disease complications [1]. Liver blood tests in isolation may not be a reliable method to identify patients at risk of cirrhosis, but together with other risk factors, they may allow the prediction of patient’s risk of developing the disease.

McLernon et al. [8, 9] took this approach and developed a prediction model and a clinical scoring tool ALFI (Algorithm for Liver Function Investigations-tool, a user-friendly version of the prediction model) to predict the risk of a liver disease diagnosis in patients who have had a liver blood test. The tool was developed in electronic health records in the Epidemiology of Liver Disease in Tayside (ELDIT) database in Scotland [10] and validated in an independent set of 19 GP practices across other areas of Scotland with data from a different time period. The purpose of ALFI is to facilitate decision-making for an immediate referral from primary to secondary care by identifying patients at high 6-month and 2-year risk of liver conditions of varying severity, ranging from fatty liver, hepatitis infections and autoimmune liver conditions to cirrhosis and disease complications.

While ALFI is able to separate out with good accuracy those primary care patients with liver blood tests who are at high risk of any liver conditions in the short term, GPs still face a problem of what to do with those patients whose test results are abnormal [11]. It is not clear which of the large number of patients with abnormal liver blood tests should be referred to specialist care, who is in serious need of preventative health intervention and who can be reassured in the long term. Presence of mild-stage liver disease such as fatty liver is becoming increasingly common, and as it may take many years for the disease to progress to severe stage, cirrhosis, or the disease may not progress at all, this may not warrant an immediate referral. While positive results on commonly ordered follow-up tests to identify viral and autoimmune liver disease after liver blood test abnormalities drive immediate referral, these cases are relatively rare. In addition to ALFI, models and risk scores (such as NAFLD fibrosis score or NFS [12], FIB-4 index [13] and the Model for End-Stage Liver Disease or MELD score [14]) that predict the stage of liver fibrosis or death in patients with existing liver disease have been developed and are in actively used in clinical decision-making. But a model that would provide information on short-, mid- and long-term risk of developing a severe stage of liver disease, cirrhosis, in patients with abnormal liver blood tests in the absence of a clear indication of liver disease, to our knowledge, does not yet exist. The use of such a model in primary care could help not only to guide referrals but also to identify those who may benefit from targeted preventative health interventions, to help behaviour change and to reassure those patients at low risk.

We aim to develop and validate a prediction model to estimate 2-, 5- and 10-year risk of cirrhosis in adult primary care patients with abnormal liver blood test results in the absence of liver disease and viral hepatitis infection.

## Objectives

The objectives of our study are to:

- Develop a prediction model to estimate the 2-, 5- and 10-year risk of cirrhosis in adult primary care patients, free from underlying liver conditions, at the time they first have an abnormal liver blood test result
- Internally validate the prediction model by examining the optimism in apparent performance and accordingly adjust the model
- Externally validate the prediction model in an independent dataset of adult primary care patients, free from underlying liver conditions, at the time they first have an abnormal liver blood test result

## Methods/design

The development of a cirrhosis risk prediction model is part of a wider study protocol (number 17_067R) that has been approved by the Independent Scientific Advisory Committee (ISAC) of the Clinical Practice Research Datalink (CPRD). The current protocol provides a publicly accessible detailed analysis plan for the development and validation of this model. The model will be developed, validated and reported in line with the Transparent Reporting of a multivariable prediction model for Individual Prognosis Or Diagnosis (TRIPOD) statement [15] and the recommended extensions for external validation in big data [16].

### Source of data

CPRD is a large primary care database containing routinely collected patient data from general practices around the UK. In terms of age, sex and ethnicity, the patients are generally representative of the UK population [17, 18]. The size of practices is above the median GP practice size in England [19]. The secondary care data for our studies comes from the Hospital Episode Statistics (HES) Admitted Patient Care data. HES data contains information on all admissions to National Health Service (NHS) hospitals in England. The Office for National Statistics (ONS) provides death registry data for CPRD patients linked to HES. The source of data in our study, the anonymised linked dataset in CPRD (GP practices using the Vision software system), currently include records of over 10 million patients from 411 practices from all regions of England [20]. The linked data is available only for England and covers approximately 75% of the CPRD practices in England.

#### Development dataset

To develop our prediction model, we will use data from all the GP practices in our dataset except practices omitted for model validation (details on the criteria for selecting the validation practices to be omitted are provided in the section below). An alternative approach for model development and validation, internal-external cross-validation (IECV) [21, 22], exists. In IECV, clusters of data are rotated between model development and validation in cycles where each cycle consists of developing the model in all except one data cluster and then validating the model in the remaining data cluster. While the advantage of IECV is that data from all GP practices is used to both develop and validate the model, the quantity of the data clusters of our primary interest, the GP practices, is very large. Choosing the approach where a number of practices are removed for a separate validation allows us to estimate how the model is likely to perform if used in a new GP practice [16].

#### Validation dataset

We will omit 30 GP practices from our model development dataset and use these to externally validate our prediction model and to understand the transportability of the model to other GP practices [16]. To ensure diversity in regions, populations, settings and case-mix variation in the validation dataset, we will select the 3 GP practices from each geographical practice region (the regions consist of the 10 historical NHS strategic health authority regions in England reported in the CPRD data), with the largest number of events over the study period for the region, for validation. The validation practices will be selected and removed from the development dataset prior to further analysis of the data.

#### Follow-up

For primary care patients in CPRD, HES-linked data are available since April 1997 and ONS-linked data since January 1998. Accordingly, the study period is between January 1998 and February 2016. Patients will enter the study at any point during this period when they fulfil the eligibility criteria (specified below). The follow-up will end on the date the patients have the study outcome (cirrhosis), the date of death, the date of leaving the GP practice, the GP practice’s last data collection date or the last date of the study period, whichever is earliest. Patients who do not have the study outcome will be censored.

### Participants

#### Inclusion criteria

All adult (≥ 18 years old) primary care patients with a primary care record of at least one abnormal liver blood test result between January 1998 and February 2016, registered with a CPRD GP practice in England (that consents to data linkage to ONS mortality and HES Admitted Patient Care data), will be considered for inclusion. The entry date for each patient will be the date of the index test result.

The liver blood tests and abnormal cut-offs that define the entry of patients to the study are listed in Table 1. In the initial investigations of liver health, a panel of different tests is ordered. The tests listed are the most common tests that may be included in the test panel. Abnormal results are defined by applying typical cut-off values currently used in primary care to the recorded units (the selected cut-off values are based on the reference ranges used in the testing laboratory of the Royal Free Hospital in London and are similar to the cut-off values used at the laboratories in hospitals in Tayside, Scotland [8], Lambeth in London and Birmingham [7]). The cut-off values have also been discussed and agreed with a hepatology consultant. When one or more of the liver blood test results come back abnormal, GPs might request a repeat test within the first 6 months of the first test. In a sensitivity analysis, we plan to assess the model performance when patients whose liver blood tests resolve (in repeat test/s, all values are within the normal threshold) are excluded. In another sensitivity analysis, we plan to derive the model coefficients for the predictors of the final model when these patients are excluded (full model development methods including bootstrapping to account for model optimism will be undertaken).

**Table 1:** Liver blood tests and abnormal values

Test	Abnormal values
Albumin	< 35 g/L
Alanine aminotransferase (ALT)	> 50 units/L
Alkaline phosphatase ALP	> 129 units/L
Aspartate aminotransferase (AST)	> 37 units/L
Bilirubin	> 21 μmol/L
Gamma-glutamyl transferase (GGT)	> 61 units/L

#### Exclusion criteria

Patients who do not fulfil the criteria for acceptable patient data quality (set by the CPRD based on registration status, recording of events, and valid age and sex [17]) will be excluded. To account for the effect of moving practice and potential delay in transfer of patient records, patients who have not yet accumulated at least 1 year of up-to-standard follow-up since their registration at the GP practice will be excluded (up-to-standard date, calculated by the CPRD, is defined as the date at which the GP practice data is deemed to be of research quality in terms of continuity of recording and the recording of deaths [17]). To avoid capturing patients with existing liver disease, patients who prior to or within the first 6 months after the index date have a record of the outcome, any other form of chronic liver disease or a condition that may indicate presence of liver disease (including a record of hepatitis B or C infection), a record of primary or secondary liver cancer and/or a record of liver transplant, will be excluded. Patients who at entry have bilirubin level > 35 μmol/L (indicating potentially visible jaundice, a possible complication of cirrhosis [9, 23]) will also be excluded.

### Outcome

The outcome in our prediction model is the first record of cirrhosis in primary care or secondary care or death records after the study entry. In primary and secondary care data, the outcome is defined as the diagnosis of cirrhosis or a cirrhosis-related complication, procedure or medication. In death records, the definition includes cirrhosis or chronic liver disease as the main cause of death and cirrhosis as a secondary cause of death. The complications and related treatments included in the outcome definition are listed in Table 2. Jaundice, although a possible complication of cirrhosis, is more often seen in connection to other conditions (such as malignancy, gall stones or sepsis) and will not form part of our outcome [24, 25]. In a sensitivity analysis, we plan to evaluate the predictive model’s performance when the outcome is defined using a narrower definition of cirrhosis (based strictly on cirrhosis as a diagnosis or cause of death). In a further sensitivity analysis, we plan to derive the model coefficients for the predictors of the final model using this narrower outcome definition (full model development methods including bootstrapping to account for model optimism will be undertaken). Code lists for the cirrhosis and its complications have been adapted from previous cirrhosis studies in CPRD [2, 26] together with hepatology and gastroenterology consultants. Our model will predict the risk of outcome at 2, 5 and 10 years from study entry.

**Table 2:** Cirrhosis complications and treatments that will be considered as a record of the outcome (cirrhosis)

Cirrhosis complication	Related treatment
Ascites (no record of cancer and excluding records for malignant ascites)	Paracentesis (procedure), spironolactone ≥ 50 mg/day (diuretic medication) (no record of cancer and excluding records for malignant ascites)
Spontaneous bacterial peritonitis (no record of cancer, no records for malignant ascites)	
Hepatic encephalopathy	
Portal hypertension or oesophageal varices (with or without bleeding)	Banding (procedure), endoscopic sclerotherapy (procedure), transjugular intrahepatic portosystemic shunt (procedure)

### Predictors

The majority of liver disease in the UK develops after prolonged exposure to lifestyle-derived risk factors [2, 3, 27]. Main risk factors include harmful alcohol consumption, obesity and type 2 diabetes (reviewed in the recent NICE guideline [4]).

Our planned candidate predictors include body mass index (BMI), diabetes, alcohol consumption and 36 other factors for routinely recorded socio-demographic characteristics, modifiable lifestyle factors, co-morbidity diagnoses, liver blood and other blood test results, medications and immunisations of patients that are accessible to the GPs and likely to be available at the index date (Table 3). Mental health conditions have been included as they may be associated with increased alcohol consumption. Inflammatory bowel disease may be linked to a liver condition, such as primary sclerosing cholangitis, that may progress to cirrhosis [28]. Our list of predictors encompasses the same/similar predictors used to develop the ALFI tool [9]. Additionally, with access to the data recorded in primary care, we are able to include those potentially important predictors, such as alcohol consumption and body mass index (BMI), that could not be included in the development of ALFI (ELDIT consists of biochemistry, secondary care, prescription and mortality data but not routinely recorded data from primary care [10]). Many of our candidate predictors coincide with the tests and measures included in the NHS health checks (routine check-up in England for adults aged 40–74 to detect early signs of stroke, kidney disease, heart disease, type 2 diabetes or dementia) and the related risk tools such as QRisk [29].

**Table 3:** Candidate predictors

Socio-demographic characteristics				
Candidate predictor	Units/categories	Definition	Variable type (number of categories)	Source data
Age	Years	Year of birth subtracted from the year of study entry	Continuous	Primary care
Sex	Male/female	As recorded	Categorical (2)	Primary care
Ethnicity	White/mixed/Asian/Black/others	As recorded (codes collapsed into the five Census categories [18])	Categorical (5)	Primary care, secondary care
Social deprivation	Index quintile	Patient Level Index of Multiple Deprivation, based on patient postcode	Categorical (5; data not accessible as continuous)	Secondary care
Lifestyle				
Candidate predictor	Units	Definition	Variable type	Source data
BMI	Index (kg/m^2^)	As recorded	Continuous	Primary care
Alcohol consumption	Non-drinker/ex-drinker/occasional drinker/moderate drinker/heavy drinker	Based on the records of daily and weekly units and drinker status codes [31] with equal classification for both sexes	Categorical (5)	Primary care
Smoking	Non-smoker/ex-smoker/current smoker	As recorded	Categorical (3)	Primary care
Use of injected drugs	Present/absent	As recorded	Categorical (2)	Primary care
Co-morbidities				
Candidate predictor	Units	Definition	Variable type	Source data
Hypertension	Present/absent	Diagnosis of hypertension or record of at least three high blood pressure readings within 1 year or at least two systolic or diastolic high blood pressure readings or prescriptions for blood pressure lowering medication within 6 months	Categorical (2)	Primary care, secondary care
Diabetes	Type 1/type 2/ uncertain type/no diabetes	Diagnosis of diabetes and type	Categorical (4)	Primary care, secondary care
Inflammatory bowel disease	Present/absent	Diagnosis of Crohn’s disease or ulcerative colitis	Categorical (2)	Primary care, secondary care
Mental illness and behavioural disorders	Present/absent	Diagnosis of schizophrenia, psychosis, bipolar disorder, another mood affective disorder, depression, anxiety or phobia, reaction to stress or adjustment disorder	Categorical (2)	Primary care, secondary care
Psoriasis	Present/absent	Primary and secondary care diagnoses, or diagnosis in one data source only and supportive information through referral, dermatologist visit, medication or positive laboratory test	Categorical (2)	Primary care, secondary care
Cardiovascular disease	Present/absent	Diagnosis of stable or unstable angina, myocardial infarction, cerebrovascular disease, peripheral arterial disease, another atherosclerotic disease, heart failure, bradycardia, tachycardia, cardiac valve disorder, endocarditis, venous thromboembolism, atrial fibrillation or cardiomyopathy	Categorical (2)	Primary care, secondary care
Cancer	Present/absent	As recorded	Categorical (2)	Primary care, secondary care
HIV	Positive/negative	As recorded	Categorical (2)	Primary care, secondary care
Co-morbidity score	Score	Charlson co-morbidity index excluding liver disease	Continuous	Primary care, secondary care
Laboratory tests				
Candidate predictor	Units	Definition	Variable type	Source data
Albumin	g/L	As recorded	Continuous	Primary care
ALP	units/L	As recorded	Continuous	Primary care
ALT	units/L	As recorded	Continuous	Primary care
AST	units/L	As recorded	Continuous	Primary care
Bilirubin	μmol/L	As recorded	Continuous	Primary care
GGT	units/L	As recorded	Continuous	Primary care
High-density lipoprotein	mmol/L	As recorded	Continuous	Primary care
Low-density lipoprotein	mmol/L	As recorded	Continuous	Primary care
Triglycerides	mmol/L	As recorded	Continuous	Primary care
Total cholesterol	mmol/L	As recorded	Continuous	Primary care
Platelet count	count/L	As recorded	Continuous	Primary care
Medications, procedures and immunisations				
Candidate predictor	Units	Definition	Variable type	Source data
Bariatric surgery	Yes/no	As recorded	Categorical (2)	Primary care, secondary care
Paracetamol overdose	Yes/no	As recorded	Categorical (2)	Primary care, secondary care
Statins	Yes/no	Prescription of statins or statins with ezetimibe	Categorical (2)	Primary care
Prescription non-steroid anti-inflammatory drugs	Yes/no	Prescription	Categorical (2)	Primary care
Opioid substitution treatment	Yes/no	Prescription for methadone or buprenorphine	Categorical (2)	Primary care
Cardiovascular drugs	Yes/no	Prescription for antiplatelet, antiarrhythmic, fibrinolytic, positive inotropic, hypertension and heart failure-related or lipid-regulating (excluding statins) drugs or beta blockers, diuretics, nitrates or warfarin	Categorical (2)	Primary care
Antibiotics	Yes/no	As recorded	Categorical (2)	Primary care
HAV vaccination	Received/not received	As recorded	Categorical (2)	Primary care
HBV vaccination	Received/not received	As recorded	Categorical (2)	Primary care
Pneumococcal vaccination	Received/not received	As recorded	Categorical (2)	Primary care
Influenza vaccination	Received/not received	As recorded	Categorical (2)	Primary care

The list of candidate predictors was developed together with public health, hepatology and gastroenterology consultants. The related code lists were developed using the definitions and algorithms available on the CALIBER Data Portal www.caliberresearch.org/portal [30] and previously published code lists [18, 31].

Since 2004, through the Quality and Outcomes Framework (QOF), GPs have been incentivised to manage selected conditions that are of major public health concern such as diabetes and obesity (although not liver disease) and as a consequence recording of some of our candidate predictors may have improved. In a sensitivity analysis, we plan to assess the predictive model’s performance when patients who enter the study before 2004 are excluded. In an additional sensitivity analysis, we plan to derive the model coefficients for the predictors of the final model when these patients are excluded (full model development methods including bootstrapping to account for model optimism will be undertaken).

### Sample size

Based on a previous work using linked CPRD data in which 5118 incident cirrhosis patients were identified in primary or secondary care during a 12-year period from 1998 to 2009 [1], we estimate that at least 426 patients per year are diagnosed with cirrhosis. Therefore, during our study period from 1998 to 2016, there will be around 6887 new cirrhosis cases (study period ends in February 2016). Nearly 50% of cirrhosis cases may be first diagnosed during emergency hospitalisation [1]. Therefore, we estimate that approximately half, or 3443, of the cirrhosis patients will have had prior indicators of liver problems, i.e. abnormal liver blood test results, in primary care prior to their cirrhosis diagnosis. As few as 3% of people with abnormal liver blood test results may be diagnosed with some form of liver disease [7]. If approximately 15% of these have cirrhosis [9], continuing our above estimate of 3443 cirrhosis patients with abnormal LFTs, then the approximate number of patients with a form of liver disease is 22,953. This will give us a population of approximately 765,111 patients (with an abnormal liver blood test result) with the estimated 3443 outcome events.

As we are planning to omit 30 GP practices for model validation, assuming all of the 411 practices will be included in our dataset and an even distribution of outcome events across practices, we will have 251 events in the validation dataset (above the estimated minimum requirement of 100 events [32]). For our development dataset, we will have 3192 events. We are interested in 39 predictor variables that may require potentially as many as 65 parameters to be estimated (counting in multiple categories and assuming each of our 14 continuous predictors requires an extra parameter for a non-linear term). This would provide our model with 49 events per variable.

As our cirrhosis outcome definition is broader than the definition used in the studies, our sample size calculation is partly based on (in these studies outcome definitions did not include cirrhosis-related deaths [2, 9, 26] and cirrhosis-related records in primary care [9]), it is likely that our sample size calculation is conservative and the events per variable in our model will be larger than the estimated. It is also possible that the distribution of events across practices may not be even, and as the three practices with the largest number of events per region are omitted for validation, it is likely that the proportion of events in the validation dataset may be larger and the proportion of events in the development dataset may be smaller than the estimated. We may also consider reducing the number of parameters to be included in the model (further details below).

### Missing data

In co-morbidity, procedure, medication and immunisation variables, we will assume that missing records are not missing data but indicate a true absence of a diagnosis, procedure, prescription or vaccination and will assign the reference category to the missing values in each variable. In case of the laboratory measures and lifestyle variables, it is likely that the values are not missing completely at random but that the missingness can be explained by the observed values of the variables in our data such as age, co-morbidities and medication variables. For instance, older patients are likely to suffer from more co-morbidities, may be likely to visit their GPs more regularly and have a more complete recording of these variables than healthy patients (the QOF scheme and NHS health checks are likely to have further amplified this pattern). Analysis of only complete cases in this context could lead to biassed estimates [33], and so we plan to use multiple imputation to impute missing predictor data [34]. Multiple imputation will be performed both in the development dataset and validation dataset prior to model development and validation. The imputation model will include all predictors and outcome data. The number of imputations will be determined by the fraction of missing data. We will use Rubin’s rules [35] to pool estimates across the imputed datasets to produce the estimates for the final model and model performance statistics. Calibration plot will be presented for only one of the imputed datasets if representative of those produced in other imputations. We will investigate the potential mechanisms of missingness and plan to perform sensitivity analysis to explore the robustness of the multiple imputation.

### Statistical analysis methods

#### Handling of predictors

Rare categories in categorical variables may be grouped together. Candidate predictors recorded as continuous measurements will be kept as continuous in the analyses. We will allow for non-linear relationships between continuous predictors and the outcome by using multivariable fractional polynomials method to select the most suitable functional form for the continuous predictors.

#### Type of model

We will use the Cox proportional hazards regression model as the follow-up time between patients varies, and we are aiming to predict the risk of the outcome in a relatively long term.

#### Predictor selection before modelling

We may reduce our pool of candidate predictors before the analyses. We may remove the variables that are not informative (all or nearly all patients have the same predictor value). Variables with large amounts of missing data may be removed if it seems likely that these data would generally not be measured at a setting where the model is intended to be used (such as certain laboratory tests) and including the variable may limit the use of the model in practice. We will also consider removing the variables that are correlated (Spearman’s rank correlation) or combining variables that are similar as they may explain some of the same variations in the outcome and not all necessary in the model. Clinical judgement and consideration of general practicality in recording or measuring the variable will be used to select variables to be removed or combined.

#### Predictor selection during modelling

Age, sex and variables (alcohol consumption, BMI and diabetes) that can be considered well-known predictors of cirrhosis will be forced into the prediction model regardless of the significance level. We will begin the modelling procedure with a full model that includes all candidate predictors and apply backward elimination to the rest of the predictors using Wald test *p* value > 0.157 (a proxy for the Akaike Information Criterion) as the elimination criteria. Multivariable fractional polynomials method will be used as part of the variable selection.

#### Calculation of predictions

To make individual predictions, we will centre the predictors by their mean and fit the model and use the Kaplan-Meier survival function to estimate the baseline hazard of cirrhosis 1 − *S*_0_(*t*)^exp(*β*1(*X*1*i − X̅*1 + *β*2(*X*2*i − X̅*2)+…)^ (probability of cirrhosis for an individual with “average” characteristics) at 2, 5 and 10 years. Full model coefficients including the baseline hazard at 2, 5and 10 years will be reported.

#### Internal validation

The apparent calibration (how well the model predictions match the observed data) will be assessed by calculating the calibration slope. Perfect calibration to the development data (calibration slope = 1) is expected. To evaluate the apparent discrimination performance (how well the model separates between individuals who develop cirrhosis and those who remain cirrhosis free), we will plot Kaplan-Meier curves for four groups defined by Cox’s centiles [36] (separation between prognostic groups), calculate Royston’s *D* statistic [37], the corresponding hazard ratio (separation between two prognostic groups defined by median linear predictor) and *R*^2^_*D*_ [37] (variation explained by the model) and Harrell’s *C*-statistic with 95% confidence interval [38] (level of discrimination).

To examine optimism in the apparent performance and internally validate the model, we plan to use bootstrapping. The model development will be repeated in 100 bootstrap samples to estimate the average optimism in the apparent *C*-statistic and apparent calibration slope. The estimate of average optimism will be subtracted from the apparent performance statistics to produce optimism-adjusted statistics for the performance of the model. To adjust the final model for optimism, we will use the optimism-adjusted calibration slope as the uniform shrinkage factor to correct the model’s beta-coefficients and re-estimate the baseline hazard. In case the number of imputations for missing data is large and makes the estimation of the uniform shrinkage factor by bootstrapping computationally impractical (impossible), we will use the heuristic shrinkage factor (*S* = [model *χ*^2^ − df]/model *χ*^2^) [39] to correct for optimism in the model.

### External validation

To investigate the model performance in new data, we will externally validate the model in the 30 practices omitted from the model development dataset. The model performance will be assessed at 2-, 5- and 10-year time points. We plan to assess the model performance in the whole dataset and, given the size of our dataset allows for a reasonable sample size (100 events), also in the following subgroups: women and men, age groups (18–39 years, 40–65 years, 65+ years), obese (BMI ≥ 30) and not obese patients, and patients who do not drink alcohol, who drink alcohol within the current guidelines (≤ 14 units/week) and those whose alcohol consumption is above the current guidelines (> 14 units/week). To understand the transportability of the model and consistency in the performance in new GP practices and regions, given there are at least 4 practices and regions with a reasonable sample size (100 events) [22], we plan to summarise the average calibration and discrimination performance across practices and regions and derive 95% prediction intervals for the performance expected in a new practice and a new region. Sample size will be considered in the evaluation and interpretation of model performance.

#### Calibration

Calibration of the model will be assessed graphically by plotting the predicted outcome probabilities against observed outcomes. Calibration plots covering tenths of predicted risk in the whole population, for men and women separately, for subgroups of age, for patient groups based on obesity and the level of alcohol use and for each region and GP practice will be presented. Calibration slope for the whole dataset, men and women separately, for subgroups of age, for patient groups based on the level of alcohol use, and for each region and GP practice will be calculated. We will use random-effects meta-analyses [40] to summarise the average calibration performance across the regions and across GP practices and estimate the 95% prediction interval for the performance expected in a new region and a new practice. Forest plots will be used to display the results of the meta-analyses.

#### Discrimination

To evaluate the model’s ability to discriminate between individuals who develop cirrhosis and individuals who remain cirrhosis free, we will calculate Royston’s *D* statistic and *R*^2^_*D*_ and Harrell’s *C*-statistic with a 95% confidence interval. The discrimination performance will be assessed in the whole population, in men and women separately, in subgroups of age, inpatient groups based on obesity and the level of alcohol use, and in each region and GP practice. Random-effects meta-analyses will be performed to summarise the average discrimination performance and estimate the approximate 95% prediction intervals for the performance expected in a new region and in a new practice. Forest plots will be used to present the results of the meta-analyses.

### Model updating

In case the calibration plots in the validation data indicate systematic over- or underestimation of risk, we plan to explore the effect of recalibration by updating the baseline hazard for the entire validation dataset or a subgroup. The baseline hazard will be updated by fitting the original model to the validation data with the linear predictor as an offset term. The updated model will then be used to estimate the updated baseline hazard at 2 and/or 5 and/or 10 years, and the calibration performance will be evaluated comparing an updated calibration plot with the original calibration plot.

## Discussion

Both patients and health care systems would greatly benefit from better understanding of patients’ risk of developing cirrhosis. With validated risk predictions guiding referrals and preventative health interventions, life-threatening disease complications and premature deaths could be avoided and the need for hospitalisation, invasive investigations and treatment reduced. To provide enough time for preventative interventions targeting modifiable lifestyle risk factors to be successful, early identification of patients at risk of developing cirrhosis is required. The development and validation of a prediction model to accurately estimate the 2-, 5- and 10-year risk of cirrhosis in adult primary care patients with abnormal liver blood test results, described in this protocol, aims to address this.

In future work, the transportability of this model to a different geographical setting (such as a new region or a country) or to a different and/or wider population (for example primary care patients with any liver function test results or all primary care patients) could be further evaluated. With input from patients, GPs, hepatologists and providers of preventative health intervention services, we would like to explore the use and benefit of this prediction model in clinical practice and decision-making and ultimately assess the model’s cost-effectiveness and impact on health outcomes.

## Abbreviations

ALFI: Algorithm for Liver Function Investigations
ALP: Alkaline phosphatase
ALT: Alanine aminotransferase
AST: Aspartate aminotransferase
BMI: Body mass index
CPRD: Clinical Practice Research Datalink
ELDIT: Epidemiology of Liver Disease in Tayside
GGT: Gamma-glutamyl transferase
GP: General practitioner
HES: Hospital Episode Statistics
ISAC: Independent Scientific Advisory Committee
MHRA: Medicines and Healthcare products Regulatory Agency
NHS: National Health Service
NICE: National Institute for Health Care Excellence
ONS: Office for National Statistics
QOF: Quality and Outcomes Framework

## References

[1] Ratib S, Fleming KM, Crooks CJ (2014). 1 and 5 year survival estimates for people with cirrhosis of the liver in England, 1998–2009: a large population study. J Hepatol.

[2] Ratib S, West J, Crooks CJ (2014). Diagnosis of liver cirrhosis in England, a cohort study, 1998–2009: a comparison with cancer. Am J Gastroenterol.

[3] Williams R, Aspinall R, Bellis M (2014). Addressing liver disease in the UK: a blueprint for attaining excellence in health care and reducing premature mortality from lifestyle issues of excess consumption of alcohol, obesity, and viral hepatitis. Lancet.

[4] National Institute for Health Care Excellence (2016). NICE guideline - cirrhosis in 16s: assessment and management.

[5] O’Sullivan JW, Stevens S, Hobbs FDR (2018). Temporal trends in use of tests in UK primary care, 2000-15: retrospective analysis of 250 million tests. BMJ.

[6] McLernon DJ, Donnan PT, Ryder S (2009). Health outcomes following liver function testing in primary care: a retrospective cohort study. Fam Pract.

[7] Lilford RJ, Bentham L, Girling A, et al. Birmingham and Lambeth Liver Evaluation Testing Strategies (BALLETS): a prospective cohort study. Health Technol Assess. 2013;17:1–134. 10.3310/hta17280. 10.3310/hta17280 PMC4781553 23834998

[8] Donnan PT, McLernon D, Dillon JF, et al. Development of a decision support tool for primary care management of patients with abnormal liver function tests without clinically apparent liver disease: a record-linkage population cohort study and decision analysis (ALFIE). Health Technol Assess. 2009;13:1–307. 10.3310/hta13250. 10.3310/hta13250 19413926

[9] McLernon DJ, Donnan PT, Sullivan FM (2014). Prediction of liver disease in patients whose liver function tests have been checked in primary care: model development and validation using population-based observational cohorts. BMJ Open.

[10] Steinke DT, Weston TL, Morris AD (2003). The epidemiology of liver disease in Tayside database: a population-based record-linkage study. J Biomed Inform.

[11] Standing HC, Jarvis H, Orr J (2018). GPs’ experiences and perceptions of early detection of liver disease: a qualitative study in primary care. Br J Gen Pract.

[12] Angulo P, Hui JM, Marchesini G (2007). The NAFLD fibrosis score: a noninvasive system that identifies liver fibrosis in patients with NAFLD. Hepatology.

[13] Sterling RK, Lissen E, Clumeck N (2006). Development of a simple noninvasive index to predict significant fibrosis in patients with HIV/HCV coinfection. Hepatology.

[14] Kamath PS, Kim WR (2007). The Model for End-Stage Liver Disease (MELD). Hepatology.

[15] Moons KGM, Altman DG, Reitsma JB (2015). Transparent reporting of a multivariable prediction model for individual prognosis or diagnosis (TRIPOD): the TRIPOD statement. Ann Intern Med.

[16] Riley RD, Ensor J, Snell KIE (2016). External validation of clinical prediction models using big datasets from e-health records or IPD meta-analysis: opportunities and challenges. BMJ.

[17] Herrett E, Gallagher AM, Bhaskaran K (2015). Data resource profile: Clinical Practice Research Datalink (CPRD). Int J Epidemiol.

[18] Mathur R, Bhaskaran K, Chaturvedi N (2014). Completeness and usability of ethnicity data in UK-based primary care and hospital databases. J Public Heal (United Kingdom).

[19] Campbell J, Dedman DJ, Eaton SC (2013). Is the CPRD GOLD population comparable to the U.K. population?. Pharmacoepidemiol Drug Saf.

[20] Clinical Practice Research Datalink/The Medicines and Healthcare products Regulatory Agency (2018). Clinical Practice Research Datalink: linked data.

[21] Royston P, Parmar MKB, Sylvester R (2004). Construction and validation of a prognostic model across several studies, with an application in superficial bladder cancer. Stat Med.

[22] Debray TPA, Moons KGM, Ahmed I (2013). A framework for developing, implementing, and evaluating clinical prediction models in an individual participant data meta-analysis. Stat Med.

[23] Beckingham IJ, Ryder SD (2001). ABC of diseases of liver, pancreas, and biliary system. Investigation of liver and biliary disease. BMJ.

[24] Mitchell J, Hussaini H, McGovern D (2002). The “jaundice hotline” for the rapid assessment of patients with jaundice. BMJ.

[25] Taylor A, Stapley S, Hamilton W (2012). Jaundice in primary care: a cohort study of adults aged >45 years using electronic medical records. Fam Pract.

[26] Fleming KM, Aithal GP, Solaymani-dodaran M (2008). Incidence and prevalence of cirrhosis in the United Kingdom, 1992–2001: a general population-based study. J Hepatol.

[27] GBD 2016 Disease and injury incidence and prevalence collaborators (2016). Global Burden of Disease Results Tool.

[28] Gizard E, Ford AC, Bronowicki J-P (2014). Systematic review: the epidemiology of the hepatobiliary manifestations in patients with inflammatory bowel disease. Aliment Pharmacol Ther.

[29] Hippisley-Cox J, Coupland C, Brindle P (2017). Development and validation of QRISK risk prediction algorithms to estimate future risk of cardiovascular disease: prospective cohort study. BMJ.

[30] Denaxas SC, George J, Herrett E (2012). Data Resource Profile: cardiovascular disease research using linked bespoke studies and electronic health records ( CALIBER). Int J Epidemiol.

[31] Bell S, Daskalopoulou M, Rapsomaniki E (2017). Association between clinically recorded alcohol consumption and initial presentation of 12 cardiovascular diseases: population based cohort study using linked health records. BMJ.

[32] Collins GS, Ogundimu EO, Altman DG (2016). Sample size considerations for the external validation of a multivariable prognostic model: a resampling study. Stat Med.

[33] Knol MJ, Janssen KJM, Donders ART (2010). Unpredictable bias when using the missing indicator method or complete case analysis for missing confounder values: an empirical example. J Clin Epidemiol.

[34] White IR, Royston P, Wood AM (2011). Multiple imputation using chained equations: issues and guidance for practice. Stat Med.

[35] Rubin DB (1987). Multiple imputation for nonresponse in surveys.

[36] Cox DR (1957). Note on grouping. J Am Stat Assoc.

[37] Royston P, Sauerbrei W (2004). A new measure of prognostic separation in survival data. Stat Med.

[38] Harrell FE, Lee KL (1984). Regression modelling strategies for improved prognostic prediction. Stat Med.

[39] Van Houwelingen JC, Le Cessie S (1990). Predictive value of statistical models. Stat Med.

[40] DerSimonian R, Laird N (1986). Meta-analysis in clinical trials. Control Clin Trials.

